# A narrative review of staff views about dementia care in hospital through the lens of a systems framework

**DOI:** 10.1177/17449871221142104

**Published:** 2022-12-29

**Authors:** Mary Duah-Owusu White, Fiona Kelly

**Affiliations:** Researcher, Ageing and Dementia Research Centre, Bournemouth University, Poole, UK; Lecturer, Division of Nursing, Queen Margaret University, Musselburgh, UK

**Keywords:** dementia, hospital, literature review, staff views, systems approach

## Abstract

**Background:**

Significant numbers of people with dementia are admitted into acute settings. They are likely to face poor health outcomes during hospitalisation. There is the need to fully understand the care provided to people with dementia through novel methods such as a systems approach (i.e. human interactions, policy, environment and equipment).

**Aim:**

The aim of this literature review is to explore hospital practitioners’ views on dementia care and to analyse findings using a systems approach.

**Methods:**

We conducted a narrative review of primary studies that examined dementia care in acute settings. We analysed a total of 33 papers using Thomas and Harden’s thematic synthesis guidelines.

**Results:**

Thirty-three papers met the inclusion criteria for the review. The findings were as follows: (1) staff-patient relationships (e.g. coping with difficult behaviour), (2) staff–family relationships (e.g. the benefits of involving families in patient care), (3) staff–staff relationships (e.g. building a robust multidisciplinary team), (4) staff–patient care decisions (e.g. decisions directly related to the patient), (5) the environment (e.g. difficulty in adjusting to the hospital environment), (6) policies (e.g. hospital bureaucratic processes) and (7) equipment (e.g. pain assessment tools).

**Conclusion:**

The paper revealed multidimensional challenges in the provision of dementia care within hospitals. We conclude that training programmes, hospital policies and processes aimed at improving outcomes for patients with dementia should adopt a systems approach which focuses on the relational, environmental, procedural and instrumental aspects of the hospital system.

## Introduction

There are increasing numbers of people with dementia in hospital. For example, the prevalence rate of dementia in hospital has been documented to be 44% on older persons’ wards in Switzerland (Zekry et al., 2009), 25% in Irish hospitals (Timmons et al., 2015), 21% on medical and surgical wards in Australia (Travers et al., 2014) and 40% in an English acute hospital ward (Sampson et al., 2009). These prevalence rates may not have included people with undiagnosed dementia on acute wards (Gordon et al., 2009). Therefore, the prevalence rate of dementia within hospitals could potentially be as high as 63% (Mukadam and Sampson, 2011). In addition to the high prevalence rates of dementia in acute hospital settings, there is also evidence that people with dementia experience poor hospital outcomes, including malnutrition (Fogg et al., 2017; Timmons et al., 2015), infections related to the urinary system following a surgical procedure (Hu et al., 2012), difficulties with activities of daily living (Timmons et al., 2015) and falls (Kasteridis et al., 2015). Some of the initiatives that have been employed to help improve dementia care include supporting people with dementia to facilitate autonomy alongside the provision of adequate assistance for their carers (National Institute for Health and Care Excellence, 2018).

Previous researchers have synthesised the views of hospital staff to understand the challenges that they face when providing care for people with dementia (Gwernan-Jones et al., 2020; Houghton et al., 2016; Moonga and Likupe, 2016; Turner et al., 2017b). Although these reviews have highlighted the numerous challenges that exist in the provision of hospital care for people with dementia, the evidence that they have provided is still insufficient. Turner et al. (2017b), for example, ruled out qualitative studies which focused on the end-of-life care pathway, Houghton et al.’s (2016) meta-synthesis excluded studies that involved the thought processes of staff and that of Moonga and Likupe (2016) comprised studies that were conducted only in medical wards. Duah-Owusu White et al. (2020) have proposed the use of a systems approach which is based on concepts obtained from Edwards (1972), Hawkins (1987) and Zecevic et al. (2007). A systems approach looks at the working relationship between the patient-family-staff triad (Duah-Owusu White et al., 2020). It also looks at the impact of hospital policies, environment and equipment on patient care (Duah-Owusu White et al., 2020). A systems approach has been usefully employed within hospital research. For example, The National Audit of Dementia has collected information on staff views regarding themes such as staffing levels and training (Royal College of Psychiatrists, 2019). A systems approach considers the complexity of hospital care comprehensively in a holistic manner. It is potentially beneficial to patient care because of its ability to mitigate against the multidimensional challenges that are faced by people with dementia in the hospital environment (Komashie et al., 2021). The research question for this literature review is therefore: ‘How can staff views help us to understand dementia care in hospital using a system approach?’

The aim of this literature review is to explore a wide range of generalist and specialist ward staff views through a systems framework. We focused on qualitative studies because we were interested in the views of staff in regards to dementia care.

## Methods

We decided to complete a narrative literature review because of the broad nature of our research topic. The search used the following words: (dement* OR Alzheimer* OR cognitive impair* OR memory los* OR confus*) AND (hospital* OR acute * OR inpatient dement*) AND (car* OR experie* OR view* OR phenomen*) AND (qualitativ*). The databases utilised for this review can be found below (Table 1).

**Table 1.** Databases used to search for literature.

Databases for the initial search (2007–2017)	Academic Search Complete, PsycINFO Complementary Index, CINAHL Complete, MEDLINE Complete, ScienceDirect, SocINDEX with Full Text, Supplemental Index, Directory of Open Access Journals, Education Source, British Library EThOS, SciELO, SPORTDiscus with Full Text, J-STAGE, SwePub, Business Source Complete, Informit Health Collection, Library, Information Science & Technology Abstracts, Environment Complete, Communication Source, Teacher Reference Center, Hospitality & Tourism Complete, Digital Access to Scholarship at Harvard (DASH), ERIC, Communication Abstracts, Informit Humanities & Social Sciences Collection, JSTOR Journals, IEEE Xplore Digital Library, Emerald Insight.
Databases for the additional search (2017–2021) were not identical to the previous ones. These databases were used because of their availability on EBSCO	CINAHL Complete, Complementary Index, Academic Search Ultimate, APA PsycInfo Directory of Open Access Journals, SocINDEX with Full Text, Supplemental Index ScienceDirect, Environment Complete, Education Source

**Figure 1.**
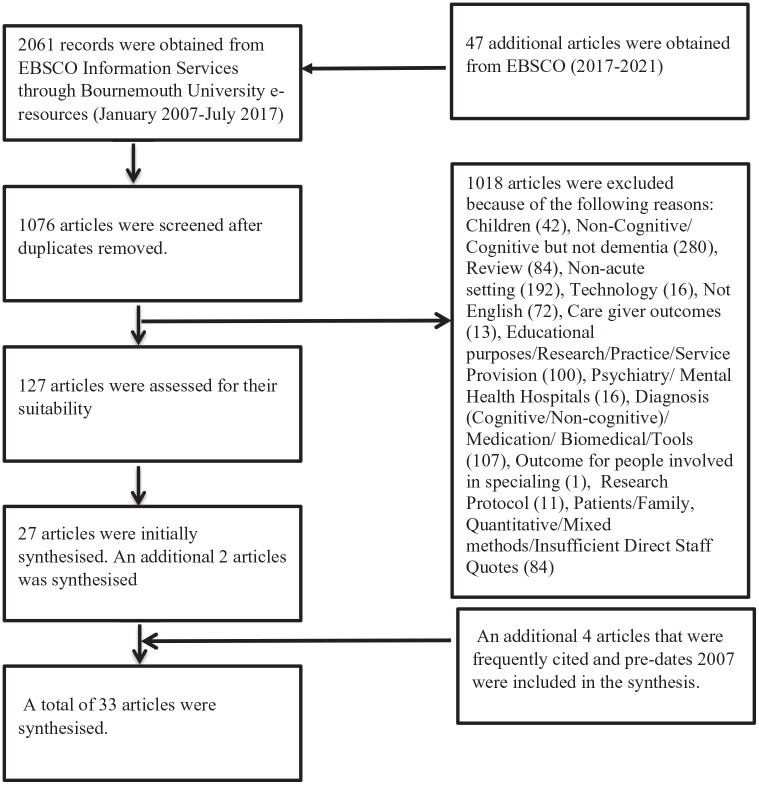
Literature search strategy.

The initial search which was conducted between 2017 and 2018 was limited to peer reviewed articles that had been published over a period of 10 years (January 2007 to July 2017). We limited the initial search to a 10-year period to access current evidence. An additional search was conducted in June 2022 using the same search terms. The time frame for the additional search was from 2017 to 2021. The qualitative review also included any frequently cited key literature that was dated before 2007. This was completed to ensure that key research evidence had been captured. Inclusion criteria for the qualitative review were: papers published in English, focused on hospital nursing care for patients with dementia or cognitive impairment and those that reported qualitative research for the dates made mention above (i.e. 2007–2021 as well as frequently cited papers before 2007). Exclusion criteria were studies that were conducted in community settings, non-qualitative research, papers written in languages other than English and research that did not focus on dementia or cognitive impairment. First of all, we initially retrieved 1076 articles after the removal of duplicates. We then screened the articles based on their titles and abstracts. We then fully read and analysed 33 papers that met the inclusion criteria (please see Figure 1).

## Analysis

The 33 papers were mainly screened and analysed by the first author. Although the authors evaluated the quality of the studies included in this review using the Critical Appraisal Skills Programme (2017), they did not exclude any of the studies on the basis of it being a poor quality paper. As Petticrew et al. (2008) point out, there is a possibility of having publication bias or skewed views when low quality studies or evidence from the grey literature is overlooked in the review of qualitative literature. Table 2 provides an overview of the studies that were analysed from 2002 to 2020 using the Critical Appraisal Skills Programme scores.

**Table 2.** Papers analysed from 2002 to 2020 with CASP scores.

Author/country	Participants/type of ward	Methods for data collection/analysis	Purpose of the study	Critical appraisal skills programme (2017)
Aita et al. (2007)/Japan	30 physicians (25 males, 5 females) from neurological, medical and surgical wards.	Interviews/Grounded Theory	To examine the factors that influences the kind of nutritional support given to people with cognitive impairment.	25
Baillie et al. (2012a)/UK	20 student nurses in a UK university who had worked on hospital wards (gender not mentioned).	Focus group/Thematic analysis	It documents the difficulties that student nurses face in dementia care.	23
Baillie et al. (2012b)/UK	20 student nurses in a UK university who had worked on hospital wards (gender not mentioned).	Focus group/Thematic analysis	It describes the techniques that are used by student nurses to provide care for people with dementia.	23
Borbasi et al. (2006)/Australia	13 nurses, 4 doctors, 3 allied health staff, 3 social workers (gender not mentioned)	Interviews/ Thematic analysis	It reports the experiences of hospital staff in relation to the provision of dementia care.	26
Bryon et al. (2010)/Belgium	21 nurses (1 male, 20 females) from geriatric, medical and palliative care wards.	Interviews/Grounded theory	It gives a staff account on the provision of nutritional support in dementia care.	27
Bryon et al. (2012a)/Belgium	21 nurses (1 male, 20 females) from geriatric, medical and palliative care wards.	Interviews/Grounded theory	It considers the practical knowledge base of nursing staff who provide nutritional support for people with dementia.	25
Bryon et al. (2012b)/Belgium	21 nurses (1 male, 20 females) from geriatric, medical and palliative care wards.	Interviews/Grounded theory	It explores the interactions between the nursing and medical team in relation to the provision of nutritional support in dementia care.	25
Chater and Hughes (2013)/UK	4 nurses, 3 healthcare assistants from medical wards.	Focus groups/Grounded theory	It documents the best approaches that can be used to enhance the knowledge base of staff involved in dementia care.	22
Cowdell (2010)/UK	25 nurses, 33 healthcare assistants from geriatric wards. 11 patients (only analysed views and observations from staff).	Interviews and Observations/Ethnography	It seeks to document the hospital care given to people with dementia.	27
Dowding et al. (2016)/UK	52 staff (nurses, doctors, healthcare assistants, additional staff) from medical, surgical, orthopaedic and specialist wards. 31 patients, 4 carers (only analysed views from staff).	Interviews, Observation, Document Analysis/Thematic Analysis	It describes how theory can be used to influence the management of pain among people with dementia.	26
Eriksson and Saveman (2002)/Sweden	12 nurses from and Accident and Emergency unit (only females)	Interviews/Thematic Analysis	To document the staff challenges in dementia care	22
Fry et al. (2015)/Australia	80 nurses (13 males, 67 females) from an emergency unit.	Focus groups/Thematic analysis	It documents the involvement of family members in the management of pain among people with cognitive impairment.	26
Fry et al. (2016)/Australia	80 nurses (13 males, 67 females) from an emergency unit.	Focus groups/Thematic analysis	It explores the experiences of hospital staff when caring for people who have cognitive impairment and are also in pain on admission.	23
Fry et al. (2017)/Australia	36 nurses (2 males, 34 females) from an emergency unit.	Focus groups/Thematic analysis	To assess the views of nurses regarding the use of different pain scales among people with cognitive impairment.	26
Fukuda et al. (2015)/Japan	50 nurses (1 male, 49 females) from a surgical and medical wards.	Focus groups/Thematic analysis	It identifies the difficulties that are encountered by staff in dementia care.	23
Handley et al. (2019)/UK	36 staff (healthcare assistants, nurses, medical staff, allied healthcare professionals and support staff) 28 patients, 2 carers (only analysed views of staff).	Interviews, review of patients medical records and observation/Thematic analysis	To identify strategies that staff use when providing dementia care	24
Hunter et al. (2017)/Canada	12 professionals (doctor, nurse, occupational health therapist, physiotherapist, social worker) from an emergency unit.	Interviews/Thematic analysis	It investigates the views of professionals regarding patient safety in dementia care.	25
Hynninen et al. (2015)/Finland	19 nurses, 9 physicians (6 males, 22 females) from a surgical unit.	Interviews/Content analysis	It documents the surgical management of people with dementia.	26
Kable et al. (2015)/Australia	21 staff (doctors, nurses, allied health staff and a specialist in the continuity of care) – only views from acute settings included in the review.	Focus group/Content analysis	It explores the views of staff regarding the discharge procedure for patients with dementia and their continuity of care.	25
Krupic et al. (2016)/Sweden	10 nurses (5 males, 5 females) from an orthopaedic unit.	Interviews/Content analysis	It describes the interactions between surgical staff and people with dementia.	25
Lichtner et al. (2016)/UK	31 nurses, 7 healthcare assistants, 8 doctors, 2 Allied health, 4 practice educators) from rehabilitation, surgical, orthopaedic and medical wards. 31 patients, 4 carers (only analysed views and observation of staff).	Interviews, Observation, Document Analysis/Thematic Analysis	It explores the management of pain among people with dementia.	27
Lichtner et al. (2015)/UK	52 staff (nurses, doctors, healthcare assistants, additional staff) 31 patients, 4 carers (only analysed views from staff).	Interviews, Observation, Document Analysis/Thematic Analysis	It explores the evaluation of pain in dementia care.	27
McPherson et al. (2016)/UK	8 nurses, 2 healthcare assistants (3 males, 7 females) from dementia wards.	Interviews/Grounded theory	It documents the challenges and strategies used in the provision of dementia care.	26
Moyle et al. (2011)/Australia	1 doctor, 9 nurses, 3 healthcare assistants from medical and surgical units.	Interviews/Thematic analysis	It elicits the views of staff in regards to the hospital care provided for people with dementia.	24
Nilsson et al. (2013)/Sweden	7 nurses, 2 doctors 1 patient, 1 family member (only analysed views from staff)	Interviews, Observations/Grounded theory	To generate a theory on the challenges that staff face when providing person-centred care for patients with cognitive impairment.	24
Nolan (2006)/Ireland	7 nurses from a geriatric unit (only females)	Interviews/Thematic analysis	It describes staff perceptions of dementia care.	26
Nolan (2007)/ Ireland	7 nurses from a gerontological unit (only females)	Interviews/Thematic analysis	It documents the views of staff in relation to dementia care.	22
Robinson et al. (2013)/UK	95 staff (nurses, doctors, social workers, legal practitioner, ambulance service team, voluntary workers, allied health staff) from hospital wards and the community (only views from acute settings included in the review).	Focus group or Interview/Thematic analysis	It documents the views of staff regarding the use of advanced care plans among people who have dementia or those on the end-of-life pathway.	23
Ryan et al. (2012)/UK	58 staff (doctors, nurses, allied health staff) from hospital wards and the community (only views from acute settings included in the review).	Focus group or Interview/Thematic analysis	It investigates the perceptions of staff in relation to the care provided for people who have dementia and have also been put on the end-of-life pathway.	22
Scerri et al. (2015)/Malta	16 nurses, 9 healthcare assistants, 8 allied health staff. (25 females, 8 males) from rehabilitation wards 10 relatives (only analysed views of staff).	Interviews/Thematic analysis	It uses appreciative inquiry to elicit the views of staff regarding useful strategies that can be employed in dementia care.	23
Spencer et al. (2014)/UK	22 staff (nurses, health care assistants, allied health staff, and an activity co-ordinator) from a specialist ward in a hospital.	Interviews/Thematic analysis	To evaluate the combined effect of using a dementia specialist and the training of staff.	27
Taneichi and Rokkaku (2020)/Japan	10 dementia nurses	Focus group interviews/Thematic analysis	It identified the experiences of dementia nurses who worked in hospitals	22
Turner et al. (2017a)/UK	3 nurses, 1 physiotherapist, 1 doctor, 3 health care assistants, 1 Ward clerk, 1 student nurse, 2 domestic assistants (10 females, 2 males) from general hospital wards	Interviews/Grounded Theory	It describes how hospital staff address the difficult situations they face in dementia care.	26

CASP: Critical Appraisal Skills Programme.

The studies included in this review were analysed using Thomas and Harden’s (2008) thematic synthesis guidelines (i.e. coding the data on a sentence-by-sentence basis as well as developing descriptive themes). Having read the 33 papers, the first author assigned codes to the findings of the qualitative papers in the following steps. The data were coded inductively (i.e. without the use of a theory) (Marks and Yardley, 2004). The coded data were then grouped under the four elements of a systems framework: interactions, hospital policy, environment and equipment (i.e. deductive coding) (Marks and Yardley, 2004). Coding was completed at the semantic level meaning that the researcher looks for a surface understanding of the dataset (Byrne, 2022). The codes were then grouped under several descriptive (inductive approach) and theoretical themes (deductive approach) (Marks and Yardley, 2004). Table 3 shows the descriptive and theoretical themes.

**Table 3.** Descriptive and theoretical themes.

Descriptive themes	Theoretical themes
Establishing rapport with the patient	Patient–staff relationships
Difficulties in communication	
Coping with difficult behaviour	
The behavioural symptoms of dementia, memory and sleeping difficulties	
Decisions directly related to the patient	Patient care decisions
Decisions related to staff	
The benefits of involving families in patient care	Staff–family relationships
The challenges of involving families in patient care	
Building a robust multidisciplinary team	Staff–staff relationships
Challenges with team working	
Inadequate training and time constraints	Hospital policies and protocols
Benefits of adequate training	
Hospital bureaucratic processes	
Differences in the interpretation of policies	
Difficulty in adjusting to the hospital environment	Hospital environment
Strategies for adjusting to the environment	
Pain assessment tools	Hospital tools/equipment
Other clinical equipment	

The descriptive themes were then grouped under the theoretical themes of the systems approach described by Edwards (1972), Hawkins (1987), Zecevic et al. (2007) and Duah-Owusu White et al. (2020). These are human relationships (i.e. patient–staff relationships, patient care decisions, staff–family relationships, staff–staff relationships), the hospital environment, policies and equipment.

## Findings

We analysed 33 qualitative papers that focused on dementia care in hospitals. Eleven papers reported findings from focus groups (Baillie et al., 2012a; Baillie et al., 2012b; Chater and Hughes, 2013; Fry et al., 2015, 2016, 2017; Fukuda et al., 2015; Kable et al., 2015; Robinson et al., 2013; Ryan et al., 2012; Taneichi and Rokkaku, 2020) and 22 papers were interviews (Aita et al., 2007; Borbasi et al., 2006; Bryon et al., 2010, 2012a, 2012b; Cowdell, 2010; Dowding et al., 2016; Eriksson and Saveman, 2002; Handley et al., 2019; Hunter et al., 2017; Hynninen et al., 2015; Krupic et al., 2016; Lichtner et al., 2015, 2016; McPherson et al., 2016; Moyle et al., 2011; Nilsson et al., 2013; Nolan, 2006, 2007; Scerri et al., 2015; Spencer et al., 2014; Turner et al., 2017a). The findings have been grouped under the following theoretical themes which were generated from a systems framework: patient–staff relationships, patient care decisions, staff–family relationships, staff–staff relationships, hospital policies and protocols, hospital environment and hospital tools/equipment.

## Patient–staff relationships

### Establishing rapport with the patient

The use of relational approaches can be helpful in the provision of care for people with dementia (Baillie et al., 2012b; Borbasi et al., 2006; Bryon et al., 2010, 2012a, 2012b; Chater and Hughes, 2013; Cowdell, 2010; Krupic et al., 2016; Nolan, 2006, 2007; Scerri et al., 2015; Spencer et al., 2014 ). The building of patient–staff relationships can rely on simple gestures like providing an extra pillow (Krupic et al., 2016) and by staff reassuring the patient (Fry et al., 2016; Krupic et al., 2016; Scerri et al., 2015). Staff can also establish rapport using the right communication skills (Baillie et al., 2012b; Lichtner et al., 2016; Nolan, 2006, 2007; Scerri et al., 2015) and by accommodating the preferences of a patient where possible (Baillie et al., 2012b; Scerri et al., 2015; Spencer et al., 2014). A group of nursing students in the United Kingdom stated that they had met the needs of a patient in the following manner:

He was absolutely fine as long as you let him help fold the pillow cases and sheets . . . He’d found the [linen] trolley at one point that somebody had left and he decided he was going to tidy them up, we thought “okay, if you really like doing that”. (Baillie et al., 2012b: 24)

It is however not always possible to meet the preferences of patients if staff have safety concerns (Handley et al., 2019). Staff also highlighted the difficulties in developing caring relationships with aggressive patients (Nolan, 2006).

It is important to pick upon the patient’s non-verbal communication (Dowding et al., 2016; Fry et al., 2016, 2017; Handley et al., 2019; Hunter et al., 2017; Krupic et al., 2016; Lichtner et al., 2015, 2016) which may be difficult to interpret (Dowding et al., 2016) but can make the patient feel safe when correctly understood (Krupic et al., 2016).

### Difficulties in communication

Staff in the studies reviewed indicated that people with dementia/cognitive impairment had communication difficulties (Borbasi et al., 2006; Eriksson and Saveman, 2002; Fry et al., 2015, 2016, 2017; Hynninen et al., 2015; Krupic et al., 2016; Lichtner et al., 2016). This made it difficult for staff to assess the desired or adverse effect of their care (Eriksson and Saveman, 2002; Fry et al., 2016, 2017), evaluate their pain (Fry et al., 2017; Ryan et al., 2012) and meet their nutritional needs (Bryon et al., 2010). Staff may resort to the use of professional opinions (Aita et al., 2007; Dowding et al., 2016; Fry et al., 2016, 2017; Lichtner et al., 2015, 2016) or provide patients with easily digestible clinical information when patients present with communication difficulties (Hynninen et al., 2015). People with dementia sometimes do not co-operate with staff because of their difficulties in understanding the medical rationale behind their treatment (Fry et al., 2016; Fukuda et al., 2015; Hynninen et al.., 2015; Krupic et al.., 2016; Moyle et al.., 2011). A staff member in Australia expressed the following:

. . . they can be pulling out their intravenous stuff . . . trying to get out of bed . . . pulling off dressings . . . pulling off their clothes . . . they can be wandering through the wards . . . If they’re in traction they can be perhaps trying to pull that out . . . twisting themselves around the bed . . . (Moyle et al.., 2011: 422)

This shows the difficulties that staff sometimes face when communicating to patients with dementia.

### Coping with difficult behaviour

In situations where patients were un-cooperative or showing behavioral symptoms of dementia such as agitation, some staff resorted to the following: the use of an alternative drug administration route (Fry et al.., 2016), 1:1 nursing (Borbasi et al.., 2006; Hynninen 2015; Moyle 2011) and the application of restraint (Borbasi 2006; Bryon et al., 2010; Eriksson and Saveman, 2002; Fukuda et al., 2015; Hunter et al., 2017; Hynninen et al., 2015; Kable et al., 2015; Moyle et al., 2011; Spencer et al., 2014). Staff sometimes delegated patient care to inexperienced students (Baillie et al., 2012a). Some hospital staff used less restrictive approaches such as offering the patient their ‘favourite food’ (Bryon et al., 2010).

### The behavioural symptoms of dementia, memory and sleeping difficulties

Staff could also face aggressive behaviour from patients (Bryon et al., 2012a; Eriksson and Saveman, 2002; Fukuda et al., 2015; Hynninen et al., 2015; Krupic et al., 2016; McPherson et al., 2016; Moyle et al., 2011). In such situations, staff prioritised the safety of the patient and other co-patients over the provision of person-centred care (Moyle et al., 2011). It was noted that some behavioural symptoms of dementia may be due to physiological conditions (Fry et al., 2016; Moyle et al., 2011) such as pain (Fry et al., 2017), the use of poor ‘management technique’ by staff (Moyle et al., 2011) and being placed in an unfamiliar hospital setting (Kable et al., 2015; Nolan, 2006). Other possible cause of the behavioural symptoms of dementia is self-frustration as a result of the patient’s inability to communicate effectively (Nolan, 2006) and the patient having an unmet need (Handley et al., 2019). Staff may find it difficult to manage patients who are nearing end-of-life and who experience the behavioural symptoms of dementia (Ryan et al., 2012). The behavioural symptoms of dementia may strain patient-patient interactions (Eriksson and Saveman, 2002; Fukuda et al., 2015). Despite this, co-patients on the ward may helpfully alert staff to the needs expressed by people with dementia (Hynninen et al., 2015) and also serve as agents of socialisation (Scerri et al., 2015). Engaging patients’ in recreational activities or diversional therapy helped them to become calm (Handley et al., 2019).

People with dementia may experience problems with their circadian rhythm and therefore stay awake all night and sleep during the day (Hynninen et al., 2015) Also, people with memory difficulties may find it difficult to give a detailed self-account of their medical history (Borbasi et al., 2006; Fry et al., 2016; Krupic et al., 2016). Hospital staff may get ‘annoyed’ if they have to repeat instructions on a regular basis because of the patient’s memory difficulties (Krupic et al., 2016). A further consequence of memory difficulty is that patients could forget to use their call bell (Lichtner et al., 2016)

It is clear from the review that some hospital staff use principles related to relational care. There is the need to develop greater consistency in hospital dementia care.

## Patient care decisions

### Decisions directly related to the patient

Staff in acute settings have to make a wide range of decisions which may involve the prioritisation of care based on the acuity of the patient’s illness (Baillie et al., 2012a; Hunter et al., 2017; Moyle et al., 2011). They also have to make decisions which are in the best interest of the patient (Bryon et al., 2010, 2012a, 2012b). Staff are mandated to optimise the flow of hospital beds (Hunter et al., 2017), have to consider treating patients conservatively (Fry et al., 2016; Hynninen et al., 2015) and also assess the factors that compound the patient’s pain (Dowding et al., 2016; Krupic et al., 2016). Staff on surgical wards were mandated to assess and address patient’s pain on a regular basis while those on medical wards use a trial and error approach (Dowding et al., 2016; Lichtner et al., 2015, 2016). Other decisions related to maintaining the safety of patients (Handley et al., 2019; Hunter et al., 2017; Kable et al., 2015; Nolan, 2007). Patients with dementia nearing the end-of-life may not always be referred to the palliative team as staff may not perceive dementia to be a terminal illness (Ryan et al., 2012). Different cultural understandings of good care may influence practice. For example, in a Japanese study, Aita et al. (2007) identified that staff may see withdrawal of nutritional support as ‘death by starvation’ (Aita et al., 2007).

### Decisions related to staff

Staff had to engage in reflective practice (Aita et al., 2007; Bryon et al., 2012a; Fry et al., 2016). Engaging in reflective practice can enhance staff decision-making processes. Other decisions involved: thinking about the financial implications of hospital care, the role of the law in patient care and meeting requests from community professionals prior to discharge (Aita et al., 2007). It was also important to involve the multidisciplinary team in patient care (Borbasi et al., 2006; Bryon et al., 2010; Dowding et al., 2016; Robinson et al., 2013; Ryan et al., 2012). The involvement of too many professionals can however result in fragmented care (Dowding et al., 2016).

From the literature review, it is clear that staff who work in acute settings are required to make numerous decisions in relation to the care of patients.

## Staff–family relationships

### The benefits of involving families in patient care

The involvement of family members may be invaluable to patient care (Borbasi et al., 2006; Bryon et al., 2010; Fry et al., 2015, 2016, 2017; Fukuda et al., 2015; Hunter et al., 2017; Hynninen et al., 2015; Krupic et al., 2016; Lichtner et al., 2016; Moyle et al., 2011; Nolan, 2006; Scerri et al., 2015; Spencer et al., 2014). Family members can help staff to identify how the patient expresses pain (Fry et al., 2016, 2017; Lichtner et al., 2016) and also to reassure the patient (Fry et al., 2015; Fukuda et al., 2015; Hunter et al., 2017; Nolan, 2006). They can also help staff to obtain an accurate account of the patient’s past medical history (Fry et al., 2015; Hynninen et al., 2015), explain medical procedures to the patient (Fry et al., 2015) and reduce the risk of litigation (Aita et al., 2007). This comment from a member of staff in Japan shows how families can help to mitigate against the risk of litigation:

I used to be concerned about possible legal problems . . . But now, I do not worry about this, because decisions regarding the patient’s end-of-life are made after much discussion with the family. I believe this decision-making process will avoid a legal problem. In my opinion, a legal problem would arise when communication between the physician and family is insufficient. (Aita et al., 2007: 6)

Despite the benefits associated with the involvement of family members in patient care, staff utilised them on ‘ad-hoc’ basis (Moyle et al., 2011).

### The challenges of involving families in patient care

The care family members provide for people with dementia may not be recognised by all staff members (Moyle et al., 2011). Moreover, the involvement of family members in hospital care can present challenges such as the need for hospital staff to address their concerns (Bryon et al., 2010; Spencer et al., 2014) and the family’s emotional well-being (Bryon et al., 2010; Nolan, 2006; Turner et al., 2017a). Staff also need to identify scenarios where hospital admissions are due to family members needing respite care (Eriksson and Saveman, 2002; Hunter et al., 2017) and staff have to recognise family members who require medical attention for themselves (Fukuda et al., 2015; Lichtner et al., 2016). There could also be conflicts such as domestic violence within the family (Lichtner et al., 2016). There can be situations where family members may not co-operate with staff due to their difficulty in understanding the rationale behind the patient’s medical care (Fry et al., 2015; Fukuda et al., 2015). Also, they can question the type of care provided for their relatives (Fry et al., 2015; Fukuda et al., 2015). This situation can be resolved by explaining the rationale behind their medical care to any family member who is prepared to listen (Fry et al., 2015).

Similarly, patients who do not understand the reasons why they are being treated may not co-operate with their family members (Fukuda et al., 2015). It is worthwhile noting that there may be differences between the opinions of family members and staff (Borbasi et al., 2006; Bryon et al., 2012a; Fukuda et al., 2015) as well as that of the patient (Aita et al., 2007; Bryon et al., 2010; Fry et al., 2015; Nolan, 2006). Also, there are some family members who may want to impose their views on the patient (Borbasi et al., 2006; Fry et al., 2015) and may not always act in the best interest of the patient (Bryon et al., 2012a; Fukuda et al., 2015; Nolan, 2006). Others may not want to be involved in certain aspects of patient care (Fukuda et al., 2015). A description of why some family members in Japan may not want to be involved with the provision of patient care is as follows:

Because patients with dementia cannot take care of a stoma by themselves, we have to ask the family to learn how to perform this task. Even if family members understand how to take care of a stoma after explanation, it is difficult for them to maintain a positive attitude, because taking care of a stoma entails disposal of feces. (Fukuda et al., 2015: 7)

Furthermore, some patients may not have any surviving family members (Fry et al., 2015; Fukuda et al., 2015; Hunter et al., 2017).

Although staff engagement with the family members of people with dementia can be useful in clinical settings, it is necessary to equip them with the skills required to manage difficult interactions and ensure best practice (e.g. making decisions which are in the best interest of the patient) when caring for people without families.

## Staff–staff relationships

### Building a robust multidisciplinary team

Team working in the acute setting can involve the following: good handovers, clinical supervision, group reflection and the use of a dementia specialist (Chater and Hughes, 2013). Factors that can help to build relationships between members of staff are as follows: provision of constructive feedback, regular tea breaks, self-care, hospital schemes that assist staff and professionalism (McPherson et al., 2016). It is also important for staff to have supportive leadership (McPherson et al., 2016; Scerri et al., 2015; Spencer et al., 2014) and enhance their self-resilience (McPherson et al., 2016; Scerri et al., 2015). There is the need to distribute work equally among team members (Hynninen et al., 2015) and also recognise the fact that staff members are an invaluable resource in the provision of care (Borbasi et al., 2006; Chater and Hughes, 2013; Dowding et al., 2016; Hunter et al., 2017; Ryan et al., 2012; Scerri et al., 2015; Spencer et al., 2014). Reliance on other members of staff in Malta is supported by the following statement:

Sometimes, there is some of the staff who is actually able to turn communication which is not in context, into something meaningful, something which can actually change the behaviour of the patient [for example a patient] who is initially aggressive, verbally aggressive . . . (Scerri et al., 2015: 1919–1920)

Specialist dementia nurses can help to build good working relationships among the patient–family–staff triad (Taneichi and Rokkaku, 2020). They can also help to facilitate discharge meetings and train inexperienced staff (Taneichi and Rokkaku, 2020).

### Challenges with team working

Staff dynamics may, however, be affected by unequal power relationships (Baillie et al., 2012a; Turner et al., 2017a) and staff thinking in silo’s rather than taking an inter-disciplinary approach (Bryon et al., 2012a, 2012b; Kable et al., 2015; Lichtner et al., 2016). There can also be professional mistrust or differences in professional opinion (Bryon et al., 2010, 2012a; Nilsson et al., 2013) as well as the adoption of the blame culture (Fukuda et al., 2015; Turner et al., 2017a). Staffing levels may be inadequate (Borbasi et al., 2006; Eriksson and Saveman, 2002; Fukuda et al., 2015; Handley et al., 2019; Lichtner et al., 2016; Spencer et al., 2014). This could lead to a gap in the provision of patient care (e.g. ideal care that staff should provide vs what they are able to offer) (Eriksson and Saveman, 2002). Also, there could be communication issues between doctors and nurses (e.g. nurses ‘feeling inferior’ and not adopting a questioning attitude) (Bryon et al., 2012b) as well as an inadequate relay of pertinent patient information to ancillary and clinical support staff (Turner et al., 2017a). This statement from a housekeeper reflects a broken down communication channel in a UK hospital: ‘I don’t get included, so I don’t get the opportunity to ask for advice’ (Turner et al., 2017a: 864). Inadequate communication between hospital and community staff can leave community staff in a dilemma as they may not know when to discontinue nutrition support for a patient with dementia who is dying (Ryan et al., 2012).

Stressful work situations (Bryon et al., 2010; Fukuda et al., 2015; Hunter et al., 2017; Hynninen et al., 2015; Kable et al., 2015; McPherson et al., 2016) and pressures in the home environment may affect the life of staff in their homes or places of work (McPherson et al., 2016). Stressful working situations can result in staff being unable to fully meet the needs of patients in their care (Hunter et al., 2017). Staff can resort to unhelpful coping behaviours such as ‘shutting-down’ (McPherson et al., 2016) or ‘passing the buck’ when faced with stressful situations (Robinson et al., 2013; Turner et al., 2017a).

Multidisciplinary team work is essential to the provision of excellent patient care. A major challenge in the acute setting is to ensure effective hospital team work when staff are under pressure.

## Hospital policies and protocols

### Inadequate training and time constraints

Hospital policies on dementia care developed by experts may not always translate into practice as senior staff may not actively implement them on the wards (Handley et al., 2019). An inadequate amount of training on dementia care (Baillie et al., 2012a; Chater and Hughes, 2013; Cowdell, 2010; Fukuda et al., 2015; Hunter et al., 2017; Hynninen et al., 2015) coupled with time constraints can translate into poor staff attitudes such as the reluctance of staff to provide nursing care for people with dementia or manage their symptoms (Baillie et al., 2012a; Borbasi et al., 2006; Cowdell, 2010; Eriksson and Saveman, 2002; Fukuda et al., 2015; Hunter et al., 2017; Krupic et al., 2016; Lichtner et al., 2016; Moyle et al., 2011; Nilsson et al., 2013; Nolan, 2007; Turner et al., 2017a). Staff may not have enough time to promote independence among acutely ill patients with dementia (Nolan, 2006). Patients can also be labelled (e.g. ‘sweet or difficult patient’) (Borbasi et al., 2006; Cowdell, 2010; Spencer et al., 2014) and staff may provide poor quality care (Borbasi et al., 2006; Eriksson and Saveman, 2002; Nilsson et al., 2013).

### Benefits of adequate training

A hands-on (Chater and Hughes, 2013) or person-centred (Spencer et al., 2014) training approach could address the training needs of staff in relation to dementia care (Chater and Hughes, 2013; Spencer et al., 2014) and improve staff attitudes (Spencer et al., 2014). A positive staff attitude in a UK hospital is reflected in this statement: ‘I’m more flexible with them [patients] now, and I try and talk the way they talk and do things differently than before like holding their hand’(Spencer et al., 2014:13). Staff training also improves patient outcomes and enhances the ability of staff to be empathetic (Handley et al., 2019). Other types of informal training that are relevant for dementia care include peer learning and the use of experiential knowledge (Handley et al., 2019).

### Hospital bureaucratic processes

Staff spend a considerable amount of time documenting patient adverse outcomes (McPherson et al., 2016) and general patient care (Lichtner et al., 2016; McPherson et al., 2016). They need to have enough time to request a prescription (Fry et al., 2016) and interpret laboratory investigations (Lichtner et al., 2016). Staff had to wait for a manager to approve the type of social care that should be available upon discharge (Hunter et al., 2017). It was also time-consuming trying to access various social care services (Borbasi et al., 2006; Kable et al., 2015). Some paperwork activities were perceived as a ‘tick the box’ process (Lichtner et al., 2015, 2016; Robinson et al., 2013). Patient care documentation was sometimes inaccurate (Lichtner et al., 2016), inconsistent (Nilsson et al., 2013) or insufficient (Dowding et al., 2016; Kable et al., 2015; Lichtner et al., 2015, 2016). Issues related to the documentation of hospital paperwork in the UK is as follows:

nurses have so many assessments now to do that [. . .], they’ve kind of lost their credibility a bit, [the Generic pain assessment form] it’s just seen as a form and a tick box exercise [. . .] it’s another thing to do and yet they have a hugely frantic day. (Lichtner et al., 2016: 9)

### Differences in the interpretation of policies

Other issues raised by staff are as follows: the differences in the interpretation of policies that guide the administration of medication (Borbasi et al., 2006; Fry et al., 2016) and the use of laboratory data (Eriksson and Saveman, 2002). There were dissimilarities in the type of guidance that was given to students on the best management of the behavioural symptoms of dementia (Baillie et al., 2012a). There were tensions generated in the acute setting as a result of compliance with the national waiting time targets (Spencer et al., 2014) and the variations in the provision of clinical care among different settings (Nilsson et al., 2013).

From the review, staff integration of policies into practice may be affected by time constraints and the differences in the interpretation of these policies. It is necessary to address the tick box practices of some staff and increase their awareness about the benefits of following the right protocols.

## Hospital environment

### Difficulty in adjusting to the hospital environment

There could be issues with the physical environment (e.g. inadequate bedside space) (Baillie et al., 2012a; Borbasi et al., 2006; Hunter et al., 2017; McPherson et al., 2016; Nilsson et al., 2013; Nolan, 2007) and ward atmosphere (e.g. bureaucracy) (McPherson et al., 2016). People with dementia may find it difficult to adjust to a new hospital environment (Baillie et al., 2012a; Borbasi et al., 2006; Hynninen et al., 2015; Krupic et al., 2016; Nolan, 2007). This may be due to reasons such as the absence of a dementia specialist to help them settle down (Kable et al., 2015; Moyle et al., 2011), the noise from a hospital monitor (Borbasi et al., 2006; Hunter et al., 2017) or a television set (Nilsson et al., 2013). Staff had to ensure that the patient is not isolated when attempting to provide a calm environment (Hunter et al., 2017). Some patients were ‘frightened’ about being in a hospital setting and therefore require reassurance on a regular basis (Krupic et al., 2016). Staff may also fail to constantly re-orientate the patient to their setting (Borbasi et al., 2006; Hunter et al., 2017). This makes it important to minimise the unnecessary movement of patients between wards (Baillie et al., 2012a; Moyle et al., 2011; Nilsson et al., 2013). However, patients have to endure multiple ward transfers (Eriksson and Saveman, 2002).

The use of a wide-range of professionals who are unknown to the patient (Eriksson and Saveman, 2002; Fukuda et al., 2015; Lichtner et al., 2016; Nilsson et al., 2013; Nolan, 2007) and the lack of recreational activities (Baillie et al., 2012a) may make it difficult for patients to adjust to their new environment. UK student nurses in Baillie et al.’s (2012a: 34) study raised their concerns regarding the absence of recreational activities in the hospital environment as follows: ‘She was in a side room but with no television, no music, nothing [. . .] it was shut off from the ward and then the room itself was shut off as well’. Patients having difficulty in adjusting to a new hospital setting may result in adverse outcomes comma e.g. falls (Fukuda et al., 2015).

### Strategies for adjusting to the environment

It is therefore important to adhere to dementia-friendly guidelines (Hunter et al., 2017; Scerri et al., 2015) and encourage the use of a patient’s personal items for the purposes of producing a familiar hospital environment (Baillie et al., 2012b; Krupic et al., 2016). There is a need to provide a specialist in dementia care (Borbasi et al., 2006; Spencer et al., 2014) and create an environment which positively reinforces the provision of excellent care (Spencer et al., 2014). Patients with dementia could also be placed next to the nurses’ station in order to ensure their safety (Hynninen et al., 2015).

Improving the hospital environment for people with dementia requires a collaborative effort between frontline staff and the management team.

## Hospital tools/equipment

### Pain assessment tools

Staff felt that it was difficult to evaluate pain among people with cognitive impairment because of the differences in articulating or describing their pain alongside the usage of a scoring system for its measurement (Fry et al., 2016; Lichtner et al., 2015). In light of this challenge, some staff resorted to pain tools which for example used ‘children’s faces’ (Fry et al., 2016). This is expressed in an Australian study as follows: ‘In the past I have used the pain scale using the children’s faces sometimes [helps]. It does depend on how impaired they are’ (Fry et al., 2016: 56). After weighing-up the advantages and disadvantages of four pain assessment tools, staff felt that the ‘Pain Assessment in Advanced Dementia tool’ was useful in managing pain (Fry et al., 2017). Tools for the documentation of pain, however, have to be used in conjunction with ‘common sense’ and empathy as they may sometimes fail to pick up the intensity of the patient’s pain (Dowding et al., 2016; Fry et al., 2017; Lichtner et al., 2015). It is worthwhile to note that the provision of adequate pain relief for the patient enables staff to carry out other nursing duties (Fry et al., 2016; Krupic et al., 2016).

### Other clinical equipment

Staff felt that patients could sometimes detach clinical equipment, for example, a peripheral line (Fukuda et al., 2015; Hynninen et al., 2015). Also, the ability of the nurse to respond to the call bells may be impeded by their low volume sound (Fukuda et al., 2015). Japanese participants in Fukuda et al.’s (2015: 8) study expressed this view as follows:

Because the nurse call button is not connected to a personal walkie-talkie system, it cannot be heard when nurses are administering care to a patient in a room far from the nurses’ station.

Patient’s personal phones were thought to be useful because they helped in the communication process by providing reassurance of direct access to their family (Hunter et al., 2017).

From the review, it is important to regularly evaluate staff views on the effectiveness of the equipment that is available in acute settings.

## Discussion

The study identified these key themes and subthemes (1) staff–patient relationships (e.g. coping with difficult behaviour), (2) staff–family relationships (e.g. the benefits of involving families in patient care) and (3) staff–staff relationships (e.g. building a robust multidisciplinary team). Other elements under the systems framework include: (4) staff–patient care decisions (e.g. decisions directly related to the patient), (5) the environment (e.g. difficulty in adjusting to the hospital environment), (6) policies (e.g. hospital bureaucratic processes) and (7) equipment (e.g. pain assessment tools).

Findings from the review indicated that there were difficulties in the interactions between patient, their families and staff. Previous research on how to develop caring relationships between patients, their family members and staff found that such interactions were underlined with the following principles: understanding the perspective of the patient and their family members, questioning routine practices, adopting flexible approaches to care and valuing exemplary care (Dewar and Nolan, 2013). Brooker (2003) and Brooker and Latham (2016) emphasise that the use of person-centred approaches which considers the perspectives and needs of a person in addition to the promotion of a supportive environment. The use of relational or person-centred approaches has been documented to have a positive impact on the well-being of the various stakeholders (patients, family members and staff) in a hospital setting (Smith et al., 2010). Despite the positive outcomes that have been documented with the use of relationship or person-centred approaches, Dewing (2004) argues that further research is needed on how to effectively integrate such approaches into everyday practice.

In view of the stressful working situations that staff in acute settings face, the use of the ‘forget-me-not’ scheme has been adopted in a number of settings to help with the provision of care for people with dementia (Wray and Lim, 2017). Schemes which are used to alert staff to needs of people with dementia should be devoid of the labels (e.g. ‘sweet or difficult patient’) that were identified in the Cowdell (2010) study.

Evidence from this review indicated that staff found it difficult to adequately manage the behavioural symptoms of dementia. McCarthy (2017) provides an example of how appreciative inquiry can be used in such situations. This review showed potential difficulties in the interactions between hospital staff and family members. The use of Jurgens et al.’s (2012) ‘cycle of discontent’ can help staff to identify difficult staff-family relationships. The cycle begins when family members scrutinise hospital care as a result of their unhappiness. This process of scrutiny may then translate into complaints if family members are able to justify their suspicions (Jurgens et al., 2012).

The concept of making decisions which are in the best interest of the patient and the use of safeguarding principles derived from capacity legislation could guide the care of people with dementia who do not have next of kin, or where there is conflict or disagreement in regard to the patient’s ongoing care.

Findings from this qualitative review provided evidence of the occurrence of silo thinking in hospitals. McCartney (2016) documents the disadvantages of silo thinking which includes the unnecessary repetition of tasks related to patient care and the potential of staff to offer conflicting professional advice. It is, therefore, important to provide collaborative team work by encouraging different members of staff to express their views, developing trustworthy relationships among the multidisciplinary team, as well as employing the use of effective and constructive communication skills (Roth and Markova, 2012).

Morey et al. (2002) conclude that training helps to foster positive team work. Furthermore, it has been found that the application of skills generated from dementia training programmes for hospital staff is useful in developing positive staff attitudes (Elvish et al., 2014; Surr et al., 2016).

Staff in this review indicated that there were differences in the interpretation of hospital policies. Principles for the effective integration and interpretation of hospital policies can be drawn from Healy’s (2012) work. This includes the following: engaging the different levels of the hospital management team, applying positive reinforcement principles, disagreeing with nonconforming staff behaviour, training of hospital staff and adapting digital systems to support the integration of hospital policies (Healy, 2012).

Principles derived from the The King’s Fund (2014) report can be used to address the structural environmental challenges that were identified in this review. In terms of making changes to the functional ward environment, Scott et al. (2003) state that this requires a multiple level approach which is driven from within. Also, it is important to tap into the value system of the hospital management team and embrace the usage of positive reinforcement principles (Scott et al., 2003).

This review documented the inappropriateness of some hospital pain assessment tools which used a scoring system in the care for people with dementia. There is the need for hospitals to use the most appropriate pain assessment tools (i.e. observational or scoring systems) for the various stages of dementia (i.e. mild, moderate and advanced). Findings from the qualitative review also indicated that patients could detach hospital equipment such as peripheral lines. Further guidance is therefore needed on how to encourage patients to co-operate with staff (Andrews, 2006) when using various hospital equipment.

## Strengths and limitations

This research has used a novel lens to analyse 33 research papers on the views of staff caring for patients with dementia in hospital of a systems approach. By doing so, it offers the opportunity to identify: relational, instrumental, environmental and procedural issues that might impact on the experiences and outcomes for people with dementia in hospital.

Limitations related to the literature review: This paper only focuses on the views of staff and, therefore, will not reflect the perceptions of people with dementia and their family members. Future research should analyse the views of patients and their family members through the lens of a systems perspective given the power differentials between patients and hospital staff. Ideally two people should have independently extracted the papers for analysis and another person should have been involved in analysis (e.g. critical friend). We did not have enough resources for this type of rigorous process.

Limitations related to the papers reviewed: As the studies included in this review were mainly the views of staff which were collected from interviews, it is possible that there may be a disconnection between what staff say they do and what they actually do. Nevertheless, policy makers may find it helpful to listen to their views. The studies analysed were conducted in high-income countries, the findings may therefore not be transferable to low-income countries. Also, the views of senior managers like the Chief Executive Officers of hospitals were excluded from the qualitative studies reviewed.

## Implications for practice

The new knowledge generated from this study is that patient care within hospitals might benefit from adopting a systems approach. This is because the systems perspective accommodates the complexity of a hospital care system/multiple factors that influence dementia care in hospital. The provision of nursing care for people with dementia may benefit from principles derived from the use of a systems approach due to its holistic emphasis. In order to improve clinical practice, dementia training programmes, hospital policy and processes may benefit from the use of a systems approach.

## Conclusion

In summary, we have illustrated the factors that affect dementia care in hospitals through the use of a systems approach. The key message is that a systems approach can potentially help to improve the care of patients with dementia. Hospital managers need to embrace this approach within their policies (e.g. resource allocation etc.). Future researchers should explore synthesising the views of patients with dementia and their carers in addition to staff views.

**Key points for policy, practice and/or research**

- A systems approach can be used to improve hospital care for patients with dementia.
- Dementia training programmes for hospital staff need to adopt a systems approach.
- Hospital policies and processes need to be based on a systems framework.
